# Supplementary material for: Anthelmintic Efficacy and Pharmacokinetics of Ivermectin Paste after Oral Administration in Mules Infected by Cyathostomins
Source: Animals (Basel). 2020 May 28;10(6):934. doi: 10.3390/ani10060934 (PMC7341313; doi:10.3390/ani10060934)
Supplement: Supplementary file 1 [file animals-10-00934-s001.pdf]

Individual FEC at each sampling times and inferential analysis between FECs at Day +14 and Day+28

**Animal identification (N. 15 animals**	**FEC (UPG)**		
	**Day0**	**Day+14**	**Day+28**
1	1500	0	0
2	3550	250	200
3	2100	150	100
4	1200	0	0
5	700	0	0
6	800	50	50
7	600	0	0
8	2550	50	0
9	1450	50	0
10	600	0	0
11	200	0	0
12	450	0	0
13	750	0	0
14	900	0	0
15	3050	150	50
T<br>(p)		0.7987<br>(0.4312)	
df		28	
standard error of difference		25.040	
